# Lack of effect of ketamine on cortical glutamate and glutamine in healthy volunteers: a proton magnetic resonance spectroscopy study

**DOI:** 10.1177/0269881111405359

**Published:** 2012-05

**Authors:** Matthew J Taylor, Eleanor R Tiangga, Roísín Ní Mhuircheartaigh, Philip J Cowen

**Affiliations:** 1 Department of Psychiatry, University of Oxford, Oxford, UK; 2 Centre for Functional Resonance Imaging of the Brain (FMRIB), University of Oxford, Oxford, UK

**Keywords:** Glutamate, ketamine, magnetic resonance spectroscopy

## Abstract

Ketamine is a *N*-methyl-D-aspartic acid (NMDA) antagonist that has been associated with temporary clinical improvement in patients with depression. Studies using magnetic resonance spectroscopy (MRS) have shown that major depression is associated with decreased levels of glutamate and glutamine (Glx) in the anterior cingulate cortex, which normalize with clinical recovery. The present study aimed to test whether a ketamine infusion would increase cortical Glx levels in healthy volunteers. Healthy volunteers received an intravenous infusion of ketamine (0.5 mg kg^−1^, *n* = 8) or saline (*n* = 9) over 40 minutes. MRS measurements were obtained at baseline, during, and at the end of the infusion. The infusion of ketamine had significant effects on mental state but there was no effect of ketamine on the levels of Glx (*F*_3,39_ = 1.70, *p* = 0.18) or glutamate (*F*_3,39_ = 48, *p* = 0.70). This study suggests that the gradual infusion of low-dose ketamine in antidepressant doses not cause changes in cortical glutamate or glutamine in healthy volunteers that are visible by proton MRS.

## Introduction

Ketamine is a *N*-methyl-D-aspartic acid (NMDA) antagonist and clinically used anaesthetic that has been employed in sub-anaesthetic doses to bring about temporary clinical improvement in patients with treatment-resistant depression (Berman et al., 2000; Zarate et al., 2006). In preclinical studies, low-dose ketamine increases glutamate outflow in the prefrontal cortex (Moghaddam et al., 1997). This is thought to reflect a loss of inhibitory tone from GABAergic interneurons on cortical pyramidal neurons, since NMDA receptors preferentially drive the interneurons in awake cortex (Homayoun et al., 2007).

Proton magnetic resonance spectroscopy (MRS) is a technique that allows safe non-invasive imaging of brain chemicals, including glutamate, *in vivo*. Using MRS, patients with major depression have been found to have decreased levels of glutamate and a combined measure of glutamate and glutamine (Glx) in the anterior cingulate cortex (ACC) (Auer et al., 2000; Hasler et al., 2007). Levels of Glx appear to normalize with clinical recovery (Bhagwagar et al., 2008; Hasler et al., 2005; Taylor et al., 2009). This raised the hypothesis that ketamine might act to normalize decreased Glx. In support of this, loading doses of ketamine have been associated with transient increases in glutamine in the ACC of healthy volunteers (Rowland et al., 2005).

The present study aimed to test whether a low-dose ketamine infusion, similar to that used in treatment studies in depressed patients, would increase cortical Glx levels in healthy volunteers.

## Methods and materials

We studied 17 healthy volunteers (11 male, 6 female; see Table 1) who were free of any Axis I diagnosis by the Standardized Clinical Interview for Diagnostic and Statistical Manual of Mental Disorders-IV (First et al., 1997) and who had not taken psychoactive medications for at least three months before commencing the study. Four volunteers (1 ketamine, 3 control) had previously taken part in a placebo-controlled research study of short-term citalopram. They were also free of any physical illness and taking no other medications except the oral contraceptive pill. After complete description of the study, all participants gave full informed written consent to the study, which was approved by the local ethics committee. Participants received an honorarium for their participation.

**Table 1.** Group characteristics. Mean values with standard deviation unless otherwise stated.

		Saline (*n* = 9)	Ketamine (*n* = 8)
Age		23.9 (1.7)	24.9 (4.7)
Gender		6 male, 3 female	5 male, 3 female
EPQ	N	7.1 (5.1)	5.6 (3.4)
	P	3.4 (3.1)	2.4 (2.2)
	E	15.1 (2.1)	15.5 (4.2)
	L	7.7 (2.3)	7.4 (3.0)
SPQ		4.9 (8.2)	6.1 (6.4)
OLIFE	UnEx	(2.1)	0.9 (1.6)
	CogDis	4.1 (4.1)	3.9 (2.2)
	IntAn	3.1 (2.4)	3.6 (1.8)
	ImpNon	5.0 (1.7)	6.6 (4.0)
CADSS	pre	0 (0)	0.1 (0.4)
	post	0.8 (1.1)	23.1 (8.6)*
HAMD	pre	0.2 (0.4)	0.1 (0.4)
	post	0.8 (1.1)	3.9 (2.7)*
YMRS	pre	0 (0)	0 (0)
	post	0.1 (0.3)	2.0 (2.7)
BPRS	pre	0.2 (0.7)	0.2 (0.7)
	post	0.2 (0.7)	9.5 (6.2)*

EPQ = Eysenck Personality Questionnaire – scales Neuroticism (N), Psychoticism (P), Extroversion (E), Lie (L); SPQ = Schizotypal Personality Questionnaire; OLIFE = Oxford-Liverpool Inventory of Feelings and Experiences – subscales; UnEx = Unusual Experiences; CogDis = Cognitive Disorganization; IntAn = Introvertive Anhedonia; ImpNon = Impulsive Nonconformity; CADSS = Clinician Administered Dissociative States Scale; HAMD = Hamilton Depression Rating Scale; YMRS = Young Mania Rating Scale; BPRS = Brief Psychiatric Rating Scale.

* *p* < 0.05, *t*-test.

Volunteers received an intravenous infusion of ketamine (0.5 mg kg^−1^, *n* = 8) or saline (*n* = 9) over 40 minutes in a parallel group design. Baseline personality ratings were measured using the Eysenck Personality Questionnaire (EPQ), Schizotypal Personality Questionnaire (SPQ) (Raine 1991), and Oxford–Liverpool Inventory of Feelings and Experiences (OLIFE) (Mason et al., 2006) in case such factors were associated with response to ketamine. Mental state effects of infusion were measured by pre- and post-infusion ratings using the Clinician Administered Dissociative States Scale (CADSS), Hamilton Depression Rating Scale (HAMD), Young Mania Rating Scale (YMRS), and Brief Psychiatric Rating Scale (BPRS). The rater was not blind to infusion type.

### Imaging

**Figure 1.**
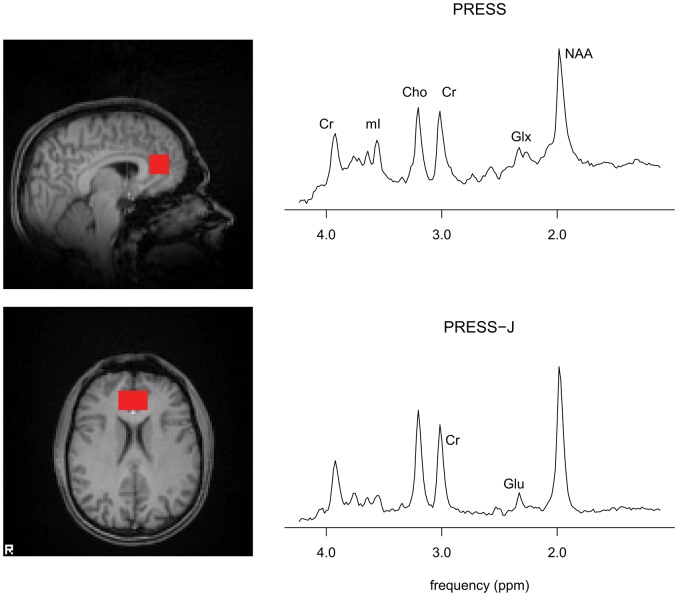
Voxel position and sample spectra from PRESS (top), PRESS-J (bottom) acquisitions. Peaks indicated for creatine (Cr), myo-inositol (mI), choline (Cho), glutamate + glutamine (Glx), *N*-acetylaspartate (NAA), and glutamate (Glu).

Scanning was performed on a 3T Varian INOVA system with a head optimized gradient coil (Tesla Engineering, Storrington, UK) and a head-only transmit/receive quadrature birdcage RF coil. MRS measurements were obtained at baseline, during (two measurements), and at the end of the infusion. PRESS and PRESS-J measurements were taken from a 30 × 20 × 20 mm voxel placed in the ACC and medial prefrontal cortex positioned manually by reference to an axial T1-weighted gradient-echo image (Figure 1). Acquisitions similarly acquired and analysed have provided good test–retest reliability for both Glx and glutamate on this system (CV < 10% in both cases, unpublished data).

PRESS data with water suppression (TE 26 ms, TR 3 s, 64 averages), and without (TE 26 ms, TR 3 s) were acquired. PRESS-J data (Hurd et al., 2004) were similarly acquired for glutamate quantitation (TE 35–185 ms, 10-ms increments; water-suppressed data, total acquisitions = 128; non-water suppressed data, total acquisitions = 16; TR = 3 s). T1-weighted structural images of whole brain were acquired with 2 mm^3^ voxel resolution. PRESS data were analysed with LCModel software (Provencher, 2001), using the non-water suppressed data for eddy current correction and water referencing, calculating metabolite concentrations for Glx, myo-inositol, choline and *N*-acetylaspartate relative to creatine in conventional fashion using metabolite basis spectra and simulated lipid and macromolecule components. A criterion was set to reject metabolite estimates with Cramér–Rao lower bound >20%; no estimates were excluded for that reason.

Measurements from the PRESS-J spectra were performed using AMARES (Advanced method for accurate, robust and efficient spectral fitting) (Vanhamme et al., 1997). PRESS-J spectra were zero-order phased, apodized with a 5-Hz Gaussian filter, and summed before analysis, and glutamate levels measured relative to creatine. FMRIB’s Automated Segmentation Tool (FAST) (Zhang et al., 2001) was employed to segment the structural brain images into grey matter, white matter, and CSF, to allow estimation of voxel composition.

### Statistical analysis

Statistical analyses were performed in SPSS version 15 using the general linear model with group and time as factors. A multivariate analysis of all available MRS measures (Glx, glutamate, myo-inositol, choline and *N*-acetylaspartate) was performed using Wilks’ lambda. The primary outcome measures of change in Glx and glutamate were tested by univariate analysis. Voxel grey matter content and age were included as covariates since an effect of voxel composition on Glx measures is well established (Mclean and Barker, 2006) and an effect of age has been described (Raininko and Mattsson, 2010). To investigate the sensitivity of the results to the use of creatine as reference, the analyses were repeated using measures referenced to tissue water. Exploratory comparisons of other measures between groups were performed by *t*-test or chi-square test as appropriate. All analyses were two-tailed.

## Results

The groups were demographically similar at baseline (Table 1). The 40-minute continuous infusion of ketamine had significant effects on mental state as reflected in increased scores on both CADSS (23.1 vs 0.8, *p* < .05) and BPRS (9.5 vs 0.2, *p* < .05) compared to placebo. HAMD scores were also significantly increased, but remained in the normal range (3.9 vs 0.8, *p* < .05).

**Figure 2.**
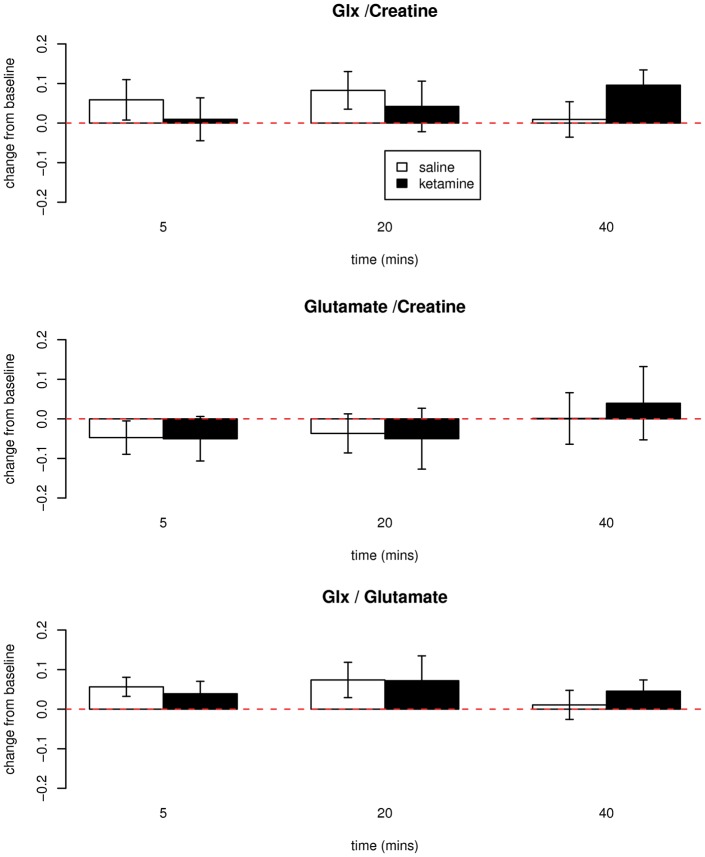
Change from baseline measurements of glutamate + glutamine (Glx)/creatine (top), glutamate/creatine (mid) and Glx/glutamate (bottom) in healthy volunteers receiving ketamine (0.5 mg kg^−1^, *n* = 8) or saline (*n* = 9) infusion over 40 minutes. Baseline Glx/Cr mean 1.69, SD 0.17; baseline Glu/Cr mean 1.76, SD 0.15. No significant differences between groups.

In contrast, there was no significant difference between groups in concentrations of cortical Glx (*F*_3,39_ = 1.70, *p* = 0.18) or glutamate (*F*_3,39_ = .48, *p* = 0.70) in the anterior cingulate cortex (Figure 2). This lack of effect of ketamine was observed whether levels were referenced to creatine or tissue water; nor were effects seen measuring Glx relative to glutamate (Figure 2). No significant effects on any other MRS measure were observed (F_15,97_ = .74, *p* = .7). The voxel contained an average 61% grey matter. Results were not sensitive to the removal of age or grey matter as covariates. No significant associations between subjective measures and MRS measures were observed.

## Discussion

This study suggests that the gradual infusion of low-dose ketamine does not cause changes visible with proton MRS in cortical glutamate or glutamine in healthy volunteers. This is in contrast to studies in animals where administration of sub-anaesthetic doses of ketamine has been shown to increase levels of glutamate in prefrontal cortex as measured by microdialysis (Moghaddam et al., 1997). However, MRS will not distinguish between glutamate in the synapse and that in other tissue compartments, for example, glia and pre-synaptic nerve terminals. Hence an increase in glutamate release without an elevation in overall tissue levels might not be detected by proton MRS, although such an effect might be demonstrated using alternative techniques such as carbon-13 spectroscopy.

As noted in the Introduction, Rowland et al. (2005) observed a small increase in glutamine levels in ACC during administration of a loading dose of ketamine (0.27 mg/kg over 10 min) but not during a two-hour maintenance infusion. It is possible, therefore, that change in glutamate/glutamine might be both transient and very sensitive to ketamine dosing regimes. We employed a regime that has been found to produce antidepressant effects in depressed patients, but the levels of ketamine achieved would probably be lower than those seen following certain target-controlled infusion techniques (e.g. Corlett et al., 2006). In addition Rowland et al. used MRS at 4T, which, unlike the present study, permitted separate delineation of glutamine and may therefore have permitted a more accurate measure of this neurochemical.

The dose of ketamine that we administered produced robust changes in mental state which were particularly apparent on the CADSS. The extent of these subjective changes was similar to that reported in other ketamine studies in healthy volunteers (Krystal et al., 1994) and also in depressed patients receiving ketamine for its antidepressant effect (Diazgranados et al., 2010). Thus ketamine administration produced clear changes in subjective mental state in the absence of measurable changes in glutamate and Glx as measured by MRS.

There are several potential limitations to this study. Changes would not have been identified if they occurred in regions outside the voxel measured. At 3T, in this study, glutamine was not resolved separately. While increased glutamine should have been reflected in increased Glx, particularly given the stable glutamate measures, at this sample size, small changes cannot be excluded. Because the effect of ketamine on glutamatergic function is thought to be mediated via GABAergic interneurons (Homayoun et al., 2007), future studies could also include gamma-aminobutyric acid (GABA) measurements. Higher field strength may allow for better differentiation and measurement of glutamate, glutamine, and GABA. It may also be possible, of course, that studies in depressed patients, who would be predicted to have low Glx levels prior to treatment, would be able to demonstrate clearer MRS effects following ketamine treatment. Ketamine treatment of depression may have effects after completion of the infusion when clinical benefits can be observed (Zarate et al., 2006).
